# A Hybrid Deep Learning Framework for Fault Diagnosis in Milling Machines

**DOI:** 10.3390/s25185866

**Published:** 2025-09-19

**Authors:** Muhammad Farooq Siddique, Wasim Zaman, Muhammad Umar, Jae-Young Kim, Jong-Myon Kim

**Affiliations:** 1 Department of Electrical, Electronics and Computer Engineering, University of Ulsan, Ulsan 44610, Republic of Korea; mfarooq229@mail.ulsan.ac.kr (M.F.S.); wasim94@mail.ulsan.ac.kr (W.Z.); muhammadumar@mail.ulsan.ac.kr (M.U.); 2 PD Technology Co., Ltd., Ulsan 44610, Republic of Korea; kjy7097@pdtech.co.kr

**Keywords:** acoustic emission signals, hybrid feature extraction, fault diagnosis, condition monitoring, hybrid deep learning model, canny edge filter, milling machine

## Abstract

This paper presents a hybrid fault-diagnosis framework for milling cutting tools designed to address three persistent challenges in industrial monitoring: noisy vibration signals, limited fault labels, and variability across operating conditions. The framework begins by removing baseline drift from raw signals to improve the signal-to-noise ratio. Logarithmic continuous wavelet scalograms are then constructed to provide precise time-frequency localization and reveal fault-related harmonics. To enhance feature clarity, a Canny edge operator is applied, suppressing minor artifacts and reducing intra-class variation so that key diagnostic structures are emphasized. Feature representation is obtained through a dual-branch encoder, where one pathway captures localized patterns while the other preserves long-range dependencies, resulting in compact and discriminative fault descriptors. These descriptors are integrated by an ensemble decision mechanism that assigns validation-guided weights to individual learners, ensuring reliable fault identification, improved robustness under noise, and stable performance across diverse operating conditions. Experimental validation on real-world cutting tool data demonstrates an accuracy of 99.78%, strong resilience to environmental noise, and consistent diagnostic performance under variable conditions. The framework remains lightweight, scalable, and readily deployable, providing a practical solution for high-precision tool fault diagnosis in data-constrained industrial environments.

## 1. Introduction

Milling cutting tools (MCTs) are indispensable in modern manufacturing, enabling the production of intricate and high-precision components across industries such as automotive, aerospace, electronics, and precision engineering [1]. These tools support complex operations such as cutting, grinding, and drilling, ensuring superior surface finishes while meeting the growing demand for cost-effective, high-performance products [2]. However, under high-speed and heavy-load operating conditions, MCTs are subjected to severe forces, friction, and thermal stresses, which result in inevitable wear phenomena such as flank wear, crater wear, and edge chipping [3,4]. This progressive degradation compromises product quality, increases machining costs, reduces tool life, and disrupts production reliability. Studies have shown that faults in key machine components, including bearings, gears, and cutting tools, account for nearly 57% of machine failures, with cutting tool defects alone responsible for approximately 20% of unplanned downtime [5,6]. Such failures not only cause extended interruptions to production but also escalate maintenance costs and inflict substantial financial losses, especially in sectors that rely heavily on high-precision milling operations. To overcome these challenges, advanced Tool Condition Monitoring (TCM) systems have been developed to optimize productivity, minimize downtime, and sustain efficiency in industrial manufacturing. In modern Computer Numerical Control (CNC) machining, such systems employ automated fault detection and tool wear monitoring to achieve reliable tool life estimation, reduce unexpected stoppages, and ensure continuous production reliability [7,8]. By enabling early detection of wear and timely intervention, TCM systems play a critical role in maintaining both the productivity and integrity of contemporary manufacturing processes [9].

Despite the progress of TCM technologies, significant challenges persist, particularly in real-world industrial environments. One of the primary limitations is the heavy reliance on large volumes of labeled data for model training, which is impractical due to the high costs, labor, and time associated with data collection and annotation [10]. Furthermore, industrial environments are inherently noisy, and these variable noise conditions introduce distortions and inaccuracies that hinder the performance of conventional monitoring methods [11]. Indirect sensing techniques, such as those based on dynamometers, accelerometers, or force sensors, are especially susceptible to external disturbances and require sophisticated preprocessing for extracting reliable features [12]. On the other hand, direct image-based approaches, while capable of delivering superior accuracy in controlled laboratory conditions, are often unsuitable for continuous real-time deployment because of their dependency on stable illumination, optics, and imaging setups [13,14]. Consequently, there is an urgent need for innovative solutions that can overcome data scarcity, remain resilient in noisy environments, and deliver scalable performance under practical industrial constraints [15,16].

The progression of fault diagnosis methods for MCTs reflects decades of innovation driven by increasing demands for precision, productivity, and automation. Early work in the 1980s and 1990s primarily investigated indirect sensor-based approaches, with vibration, acoustic emission (AE), and cutting force signals explored as indicators of tool wear [17,18]. Cutting force signals, in particular, gained prominence due to their stability and direct correlation with tool–workpiece interactions. However, such methods were limited by interference from noise, the costs of sensor installation, and the requirement for specialized instruments such as dynamometers. In the 2000s, sensor fusion strategies were introduced, combining signals such as current, force, and vibration to improve detection robustness [19,20]. Li et al. [21], for example, demonstrated the feasibility of using motor current sensors for real-time tool monitoring without the need for external instrumentation, thereby improving industrial practicality. Nevertheless, indirect methods remained highly sensitive to disturbances and involved computationally intensive preprocessing, motivating the search for more efficient alternatives [22,23].

By the 2010s, advances in machine vision systems and image processing techniques had enabled direct approaches to tool wear measurement. Researchers applied edge detection, segmentation, and texture analysis to quantify wear with high accuracy. Bagga et al. [1] proposed an edge-detection-based online wear measurement approach that achieved performance comparable to optical microscopy while minimizing machining interruptions. Around the same time, deep learning architectures, particularly Convolutional Neural Networks (CNNs), emerged as transformative tools, replacing handcrafted feature engineering with automated feature extraction and classification [24,25]. CNNs achieved remarkable performance in wear detection, classification, and segmentation tasks. However, their reliance on local receptive fields limited their ability to capture global contextual dependencies, making them less effective in complex scenarios involving overlapping or subtle wear features [26].

The 2020s marked a paradigm shift in TCM with the adoption of attention-based architectures, particularly Vision Transformers (ViTs), which leverage self-attention mechanisms to capture both local and global feature relationships [27,28]. ViTs demonstrated substantial improvements in classification accuracy and robustness, particularly in fault detection and tool wear recognition tasks [29]. Recent contributions (2023–2025) have further advanced this area, with hybrid CNN-Transformer frameworks showing superior performance by combining the strengths of convolutional feature extraction with global context modeling [30,31,32]. Parallel research has addressed noise-robust preprocessing strategies for AE and vibration signals, as well as domain adaptation and few-shot learning techniques for mitigating data scarcity [33,34]. Nonetheless, limitations persist: many current approaches still degrade significantly under high levels of industrial noise, depend heavily on large annotated datasets, or lack effective feature fusion strategies for leveraging complementary representations. Recent work in robust fault-tolerant control, such as the fuzzy adaptive scheme proposed by Bounemeur and Chemachema [35], demonstrates resilience to nonlinear actuator and time-varying sensor faults using online adaptation and Lyapunov stability analysis. While these approaches focus on stabilizing nonlinear control systems, the present study advances fault diagnosis by targeting robustness at the signal representation and feature learning levels through logarithmic CWT preprocessing, CNN–ViT fusion, and ensemble classification.

In light of these challenges, this study proposes a novel hybrid deep learning framework for milling tool fault diagnosis that integrates ResNet-18 and ViT architectures to exploit their complementary strengths. The framework employs logarithmic Continuous Wavelet Transform (log-CWT) to transform vibration signals into scalograms, enhancing resolution in critical low-frequency regions, while Canny edge filtering is used to suppress noise and emphasize salient features. ResNet-18 captures fine-grained local spatial features through hierarchical convolutional layers, whereas ViT captures long-range dependencies and global contextual information via multi-head self-attention. To further enhance robustness, extracted features are normalized and fused before being classified using a stacking ensemble comprising base learners such as Support Vector Machine (SVM), logistic regression, and a shallow Multilayer Perceptron (MLP), with a meta-learning strategy to optimize predictive accuracy.

### Contributions

The novelty and main contributions of this study include.

1. Logarithmic CWT scalograms enhanced with Canny edge filtering are introduced, providing clearer fault-relevant patterns and reducing intra-class variation compared to conventional CWT representations.
2. A hybrid dual-branch feature extraction framework is proposed, where ResNet-18 captures local features and the ViT models global dependencies, ensuring complementary and comprehensive feature learning.
3. A feature fusion and stacking ensemble classification strategy is designed, integrating linear SVM, logistic regression, and a shallow MLP with validation-guided weighting, significantly improving robustness under noisy and variable operating conditions.
4. The framework is validated on real-world vibration datasets involving milling cutting tools, gears, and bearings, achieving superior diagnostic accuracy and demonstrating practical scalability.
5. The contributions advance beyond existing CNN- or ViT-only approaches by providing a lightweight yet highly robust diagnostic solution that balances feature diversity, adaptability, and real-world deployability.

The manuscript is structured as follows. Section 2 details the methodology, outlining the key components of the framework. It also provides the technical background necessary for understanding the proposed approach. Section 3 presents the results and performance evaluation, starting with the experimental setup and dataset details, followed by an analysis of the proposed method’s effectiveness and a comparison with recent models. Finally, Section 4 concludes the study, summarizing key findings and suggesting future research directions.

## 2. Proposed Method for Fault Diagnosis in Milling Machines

**Figure 1:**
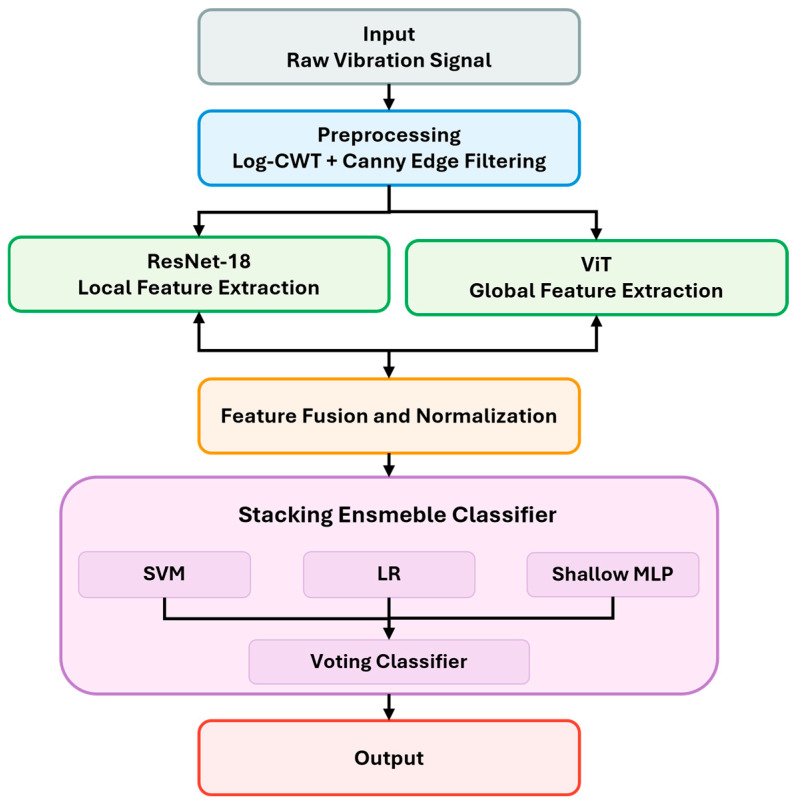
High-level block diagram of the proposed hybrid deep learning framework.

**Figure 2:**
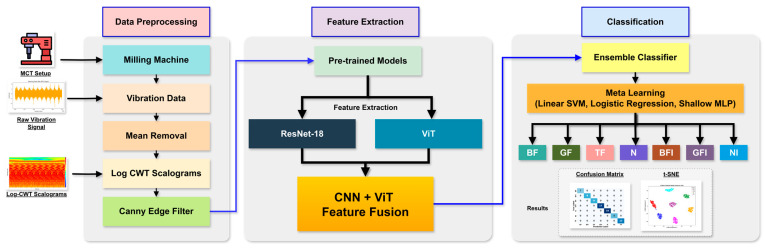
The flowchart illustrates the proposed method for fault diagnosis in milling machines.

This study introduces a novel hybrid framework that integrates ViTs and CNNs to overcome the challenges of limited datasets and noisy environments in fault diagnosis for milling machines. The block diagram of the proposed hybrid framework has been illustrated in Figure 1, where each module has been carefully designed. Conventional deep learning models frequently suffer from overfitting and limited generalization when training data is scarce, and their robustness decreases significantly in industrial conditions where background noise is unavoidable. To address these issues, the proposed method combines advanced signal preprocessing, log-CWT-based feature transformation, complementary feature extraction via CNNs and ViTs, feature fusion, and a stacking ensemble classifier, as illustrated in Figure 2. Each module has been carefully designed to enhance feature discriminability, improve prediction accuracy, and maintain computational efficiency, thereby providing a scalable and reliable solution suitable for real-world industrial applications.

**(1)**  **Data Preprocessing**

The data preprocessing pipeline begins with the acquisition of vibration signals from a milling machine during different fault conditions. Raw signals are first normalized and subjected to mean removal to eliminate baseline offsets and low-frequency drifts that could bias subsequent feature extraction. The preprocessed signals are then transformed into log-CWT, which provide a time–frequency domain representation of the non-stationary signals. Unlike standard CWT, logarithmic scaling enhances resolution in low-frequency bands that are often critical for detecting fault-related transients, while compressing higher frequencies to reduce redundancy. This transformation highlights localized bursts of energy associated with tool wear and incipient faults. To further improve robustness, a CEF is applied to the scalograms, sharpening boundaries of significant energy regions while attenuating background noise. These preprocessing steps work together to ensure that the downstream learning modules operate on clean, discriminative, and noise-resilient representations, thereby enabling reliable fault detection even under challenging manufacturing conditions.

**(2)**  **Feature Extraction**

The feature extraction module integrates the strengths of ResNet-18 and ViTs, providing dual-branch architecture. The ResNet-18 branch extracts fine-grained local spatial features through hierarchical convolutional filters and residual connections, capturing wear signatures, micro-cracks, and other localized abnormalities in scalograms. Meanwhile, the ViT branch employs a multi-head self-attention mechanism to model global dependencies across the entire scalogram, effectively capturing long-range contextual relationships that CNNs alone often overlook. The combination of local and global perspectives ensures that the model can detect both subtle and complex wear patterns across different mechanical components, including bearings, gears, and milling cutters. This dual-path design significantly enhances fault recognition accuracy and resilience to variations in operating conditions.

**(3)**  **Feature Concatenation**

To maximize the diagnostic value of extracted representations, the features obtained from ResNet-18 and ViT are passed through normalization and alignment layers before being fused. The fusion strategy is based on concatenation followed by linear projection, which integrates local spatial details with global contextual features into a unified high-dimensional representation. This enriched feature space enables the model to capture diverse fault characteristics more effectively than single-branch architecture. By aligning distributions and reducing redundancy, the fusion step ensures that complementary information from CNN and Transformer pathways is preserved, leading to a more holistic characterization of fault signatures. The fused features are then transferred to a fully connected artificial neural network (ANN) classifier, which acts as an intermediary stage to refine the decision space and provide discriminative embeddings for ensemble classification.

**(4)**  **Classification**

The final classification module employs a meta-learning stacking ensemble that operates on the fused feature embeddings. Instead of relying on a single classifier, the ensemble integrates multiple complementary base learners, including SVMs, logistic regression, and shallow MLPs. Each base learner provides a different decision perspective, capturing linear, probabilistic, and nonlinear relationships. The outputs of these learners are combined through a validation-guided weighted voting scheme, where more reliable classifiers are given greater importance and weaker predictions are suppressed. This strategy significantly improves diagnostic robustness, reduces misclassification under noise, and ensures adaptability to unseen fault patterns. The design is lightweight, computationally efficient, and capable of near real-time operation, making it deployable in industrial environments where scalability and robustness are critical.

### 2.1. Logarithmic Continous Wavelet Transform

Log-CWT is a vital technique for analyzing non-stationary signals, such as vibration data from machines, where signal frequencies evolve dynamically over time. This method plays a crucial role in machine condition monitoring by capturing subtle changes in signal patterns that indicate faults or mechanical wear. By decomposing signals into wavelets—small oscillating functions scaled logarithmically to focus on specific frequency ranges and shifted over time to capture temporal variations, the log-CWT provides a detailed time-frequency representation of the signal’s behavior [36]. Mathematically, the CWT of a signal x(t) at a given scale a, and translation b is defined as,

(1) $$W_{x}\left(a,b\right)=\int_{-\infty }^{\infty }x\left(t\right)\cdot \psi \;*\;\left(\frac{t-b}{a}\right)dt$$

where $W_{x}\left(a,b\right)$ represents the CWT coefficient, where a is the scale parameter for frequency resolution, and b is the translation parameter for time. The wavelet ψ acts as a localized filter, scaled and shifted to extract time-frequency features. While CWT provides robust time-frequency representation, its uniform scaling limits resolution in low-frequency regions.The log-CWT addresses this limitation by introducing logarithmic scaling, enhancing the resolution at lower frequencies. It is expressed as;

(2) $$W_{x}\left(log\;a,b\right)=\int_{-\infty }^{\infty }x\left(t\right)\cdot \psi \;*\;\left(\frac{t-b}{\log a}\right)dt$$

Here, the scale parameter a is replaced with loga allowing the transform to provide finer details at lower frequencies, which are often critical for detecting faults in vibration signals.

**Figure 3:**
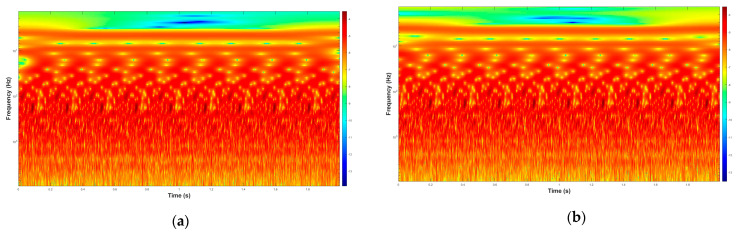
Samples of log-CWT scalograms, (**a**) Tool fault and (**b**) Gear fault from the vibration signals.

In this study, log-CWT was applied to vibration data from the MCT dataset to generate scalograms, offering detailed time-frequency visualizations. These scalograms display time on the *x*-axis, frequency on the y-axis, and color intensity representing the amplitude of log-CWT coefficients, as shown in Figure 3. The amplitude highlights the energy distribution of vibration signals across time and frequency, enabling a comprehensive analysis of machine behavior. Under normal operating conditions, scalograms exhibit stable energy patterns within expected frequency ranges, indicating smooth machine performance. In contrast, faulty conditions introduce anomalies, such as sharp energy bursts or irregular patterns, suggesting mechanical stress, impacts, or crack propagation, which act as early indicators of potential faults. The log-CWT provides distinct advantages over traditional CWT for vibration signal analysis. Its logarithmic scaling enhances resolution in low-frequency regions, capturing critical fault features like bearing or gear wear that may be overlooked by CWT. Additionally, the log-CWT balances time-frequency representation, making it better suited for signals with wide-ranging frequency components. These strengths enable more accurate detection of subtle and evolving fault signatures, making the log-CWT an invaluable tool for machine condition monitoring and early fault detection. For comparison, conventional CWT offers a uniform scale that often compresses fault-related low-frequency features, whereas log-CWT enhances resolution in these regions and captures subtle transients more effectively. This advantage justifies the use of log-CWT in the present study for robust fault feature extraction under noisy industrial conditions.

### 2.2. Canny Edge Filter

After generating the log-CWT scalograms, the next important step in preprocessing is applying the Canny Edge Filter (CEF) to enhance important structural details. The canny edge detection algorithm is widely used in image processing for identifying edges, and in the context of fault diagnosis, it helps highlight the most significant features in the scalogram by detecting boundaries between different frequency components [37,38]. The CEF operates in multiple steps to detect edges efficiently. First, the Gaussian filter smooths the scalogram to reduce noise, ensuring that only meaningful frequency transitions are captured. Mathematically, the Gaussian filter is applied as.

(3) $$G\;\left(x,\;y\right)=\frac{1}{2\pi \sigma^{2}}\;e^{-\frac{x^{2}+y^{2}}{2\sigma^{2}}}$$

where σ is the standard deviation controlling the degree of smoothing. This step prevents small variations from being falsely detected as edges. Next, the gradient magnitude and direction of the image are computed using Sobel operators in both the x and y directions:

(4) $$G_{x}=\;\frac{\partial \;S_{log}\;(x,y)}{\partial \;x}\;,\;{\;G}_{y}=\;\frac{\partial \;S_{log}\;(x,y)}{\partial \;y}$$

where $S_{log}\;(x,y)$ is the logarithmic CWT scalogram. The overall gradient magnitude (M) is then given by.

(5) $$M\left(x,y\right)=\surd (Gx^{2}+{Gy}^{2})$$

Applying the canny edge filter to the log-scaled CWT scalogram enhances the most critical frequency transitions, making it easier for deep learning models, CNN, and ViT to extract meaningful features. By emphasizing key spectral structures, this preprocessing step significantly improves the accuracy and reliability of fault classification in milling machine diagnostics.

### 2.3. Vision Transformer

ViTs represent a groundbreaking approach to computer vision, leveraging the transformer architecture originally designed for natural language processing. Unlike CNNs, which rely on convolutional layers to process images, ViTs divide images into fixed-size patches, flatten them, and treat these patches as input tokens for a transformer encoder. This design allows ViTs to capture global relationships between image patches using self-attention mechanisms. ViTs have demonstrated state-of-the-art performance in tasks such as image classification, particularly when pre-trained on large datasets and fine-tuned for specific downstream applications [39].

The key advantages of ViTs over traditional CNNs include their ability to model long-range dependencies and their adaptability across diverse datasets. Unlike CNNs, which rely on local receptive fields, ViTs capture global relationships within a single layer, enhancing feature learning. However, ViTs typically require large-scale pre-training data to achieve optimal performance. In cases with limited data, fine-tuning pre-trained ViTs or using hybrid approaches that incorporate CNN-based feature extraction can improve their effectiveness [40]. Their modular design, inspired by transformer architectures in NLP, allows flexibility in adjusting model complexity based on data availability, making them a viable option even in data-constrained scenarios [41]. The mathematical steps of ViTs can be summarized as follows:

1. **Patch embedding:** The patch embedding step converts an image into a sequence of patches and em-beds them in a high-dimensional space. The image $x\in {\mathbb{R}}^{H\;\times \;W\;\times \;C}$ where H is the height of the image, W is the width of the image, and C represents the number of the channels. The image is divided into N patches. The mathemati-cal formula for the number of patches is given below.
  (6) $$N=\frac{H\;*\;W}{P^{2}}$$
  In Equation (6), N represents the number of patches, H and W denote the height and the width of the image, whereas P represents the patch size. Each of these patches is flattened into a vector and linearly projected to a latent dimension D. Mathematically, it can be defined as.
  (7) $$z_{0}=\left[\;x_{class};\;x_{P}^{1}E;\;x_{P}^{2}E;\;x_{P}^{3}E;\ldots \ldots \;{x\;}_{P}^{N}E\;\right]+E_{pos}$$
  In Equation (7), $x_{class}$ represents the special token for classification, E is the learnable embedding matrix that maps each patch to the latent dimension, whereas $E_{pos}$ is the positional embedding, added to encode the spatial position of each patch. $x_{P}^{1}$, $x_{P}^{2}$ denotes the flattened vector corresponding to the first and second patches of the input image, respectively.
2. **Transformer encoder:** The transformer encoder processes the patch embeddings using self-attention and MLP layers, learning rich contextual representations. The input sequence is processed through L layers of transformer blocks, where each block includes multi-head self-attention, denoted by MSA, and a feedforward neural network such as MLP. Mathematically, it can be expressed as.
  (8) $$MSA(X)=Concat\;\left(\left({head}_{1},{head}_{2}\ldots \;{head}_{h}\right)\right)+z_{l-1}$$
  where each attention head computes:
  (9) $${head}_{i}=\;softmax\;\left(\frac{Q\;*\;K^{T}}{{\sqrt{d}}_{k}}\right)\;*\;V$$
  Q, K, and V are query, key, and value matrices derived from the input embeddings.
  (10) $$MSA(X)=ReLU\;\left({XW}_{1}+b\right)W_{2}+b_{2}$$
  These components are combined with residual connections and normalization, ensuring stable and efficient learning. The resulting contextual embeddings enable the ViT to capture both local and global relationships for fault diagnosis.

1. **Classification head:** The classification head of the ViT processes the output embeddings from the transformer encoder to produce the final classification result. It starts with Layer Normalization to improve stability and training, followed by the extraction of features from the classification token x-class learned during encoding. These features are then passed through a Fully Connected (FC) Layer, mapping them to the required output classes and generating prediction probabilities. This design ensures the classification token effectively captures relevant global information from the input image [42].

**Figure 4:**
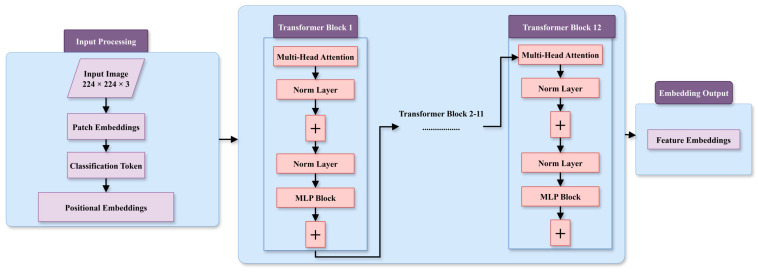
Working of vision transformer.

The proposed fault diagnosis method utilizes a ViT architecture, as shown in Figure 4, to effectively extract local and global features from input scalograms. The process begins with dividing the input image (224 × 224 × 3) into fixed-size patches, which are transformed into patch embeddings. A classification token is added to capture global information, and positional embeddings are incorporated to retain spatial relationships. These embeddings are passed through a series of transformer blocks, each consisting of multi-head attention mechanisms to capture dependencies across patches, normalization layers for stability, and MLP blocks for feature refinement. The final transformer block outputs feature embeddings that encapsulate complex spatial dependencies within the input data. These embeddings are then processed by the classification head to predict fault categories with high accuracy, demonstrating the model’s robustness and effectiveness for fault diagnosis tasks. The architectural summary of the ViT for the current work is presented in Table 1.

### 2.4. Features Pool

**Figure 5:**
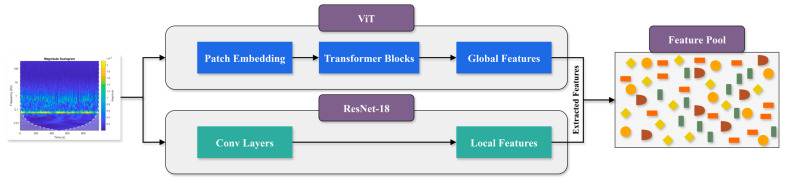
Feature pool for ResNet-18 and ViT features.

Feature extraction is performed using ResNet-18 and ViT, each capturing different aspects of the log-CWT scalograms. The fusion of these feature sets as shown in Figure 5 and Table 2 ensures a more comprehensive understanding of the fault characteristics. Mathematically, if $F_{ResNet-18}$ and $F_{ViT}$ are the feature vectors extracted from CNN and ViT, the fused feature representation is given by:

(11) $$F_{f}=F_{ResNet-18}⊕\;F_{ViT}$$

where ⊕ represents feature concatenation. To ensure balanced feature contribution, Min-Max Scaling is applied to normalize the fused vector:

(12) $$F_{f}=F_{Resnet-18}⊕\;F_{ViT}$$

where $F_{min}$ and $F_{max}$ are the minimum and maximum values of the feature set. This step prevents feature dominance and improves classification performance [43]. The fused feature vector is then fed into a classifier for fault detection, utilizing both local spatial patterns from CNN and global contextual understanding from ViT, resulting in improved accuracy and robustness in milling machine diagnostics. The concatenated feature vector $F_{f}$ is normalized using Min-Max scaling, which ensures balanced contribution by restricting each feature component to a comparable numerical range [0, 1]. This prevents domination of one feature source i.e., CNN over the other ViT. Formally, for feature $f_{i}$:

(13) $$f_{i}^{\prime }=\frac{f_{i}-f_{min}}{F_{max}-F_{min}}$$

### 2.5. Ensemble Classification with Meta-Learner

In the current study, a stacking ensemble learning (SEL) approach is adopted as the final classification module for fault diagnosis, replacing the conventional single classifier. This decision is driven by the need to improve generalization, reduce misclassification in overlapping fault categories, and leverage diverse learning strategies. After extracting features from the vibration scalograms using a pretrained ResNet-18 and ViTs, the fused features are passed to multiple base-level classifiers, each offering a unique perspective on the classification task. The ensemble takes the fused feature vector (ResNet-18 + ViT) and feeds it to three base learners-Linear SVM (kernel = ‘linear’), Logistic Regression (solver = ‘lbfgs’), and a shallow MLP (hidden_layer_sizes = (64,)). Each produces an independent prediction, which is combined by a validation-guided, meta-learned weighted voting module (“Meta Learning Logic Box”). This two-stage meta-ensemble emphasizes consistently reliable learners and down-weights weaker ones, improving robustness to individual model bias and yielding the final prediction. Mathematically, if F represents the fused feature vector and B1, B2, …, Bn represent the base learners, the meta-learner M produces the final prediction as:

(14) y^ = M(B1(F),B2(F),…,Bn(F))

**Figure 6:**
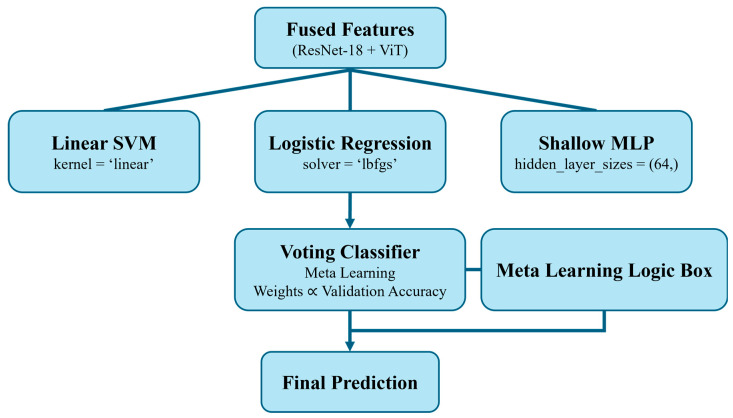
ResNet-18 + ViT Feature Fusion with Meta-Learned, Validation-Weighted Voting (SVM, Logistic Regression, Shallow MLP) for final prediction.

This architecture facilitates effective learning from multiple decision boundaries and improves fault separability, especially in cases where class distributions are imbalanced or highly overlapping. The stacking ensemble is particularly beneficial in industrial fault diagnosis tasks, where real-world data may exhibit noise, non-linearity, and high variability in fault signatures. As depicted in Figure 6, the process begins with feature extraction using ResNet-18 and ViT, followed by the fusion of features and classification through the stacked ensemble. The integration of diverse learners and a robust meta-learner enhance the model’s capability to classify subtle and overlapping fault categories, making it well-suited for real-world applications in intelligent predictive maintenance. In the stacking ensemble, each base classifier is assigned a weight proportional to its normalized validation accuracy, such that classifiers with higher reliability contribute more strongly to the final decision. This validation-weighted voting scheme contrasts with simple majority voting, where all classifiers are equally weighted. Comparative experiments confirm that validation-weighted voting yields 2–4% higher accuracy than simple majority voting and 4–7% higher than the best individual classifier, demonstrating its effectiveness in improving robustness under noisy and limited data conditions.

Unlike conventional CNN-only or Transformer-only frameworks, the proposed design integrates log-CWT preprocessing, ResNet-18 feature extraction, and ViT global attention, combined with a meta-learning stacking ensemble. This ensures improved robustness in noisy industrial environments and limited-data regimes, which are less frequently addressed in existing hybrid fault diagnosis studies. Our results demonstrate that the integration is theoretically justified (CNN local + ViT global features) where feature fusion improves classification accuracy by over single-branch models.

## 3. Results and Performance Evaluation

The proposed method is validated using vibrational data from a real-world milling machine, focusing on fault detection and classification. Its fault detection capability is first evaluated against traditional time-domain indicators, highlighting its advantages. Subsequently, its performance in fault classification is compared with state-of-the-art methods, demonstrating superior accuracy in identifying various fault types.

### 3.1. Experimental Setup and Data Acquisition

**Figure 7:**
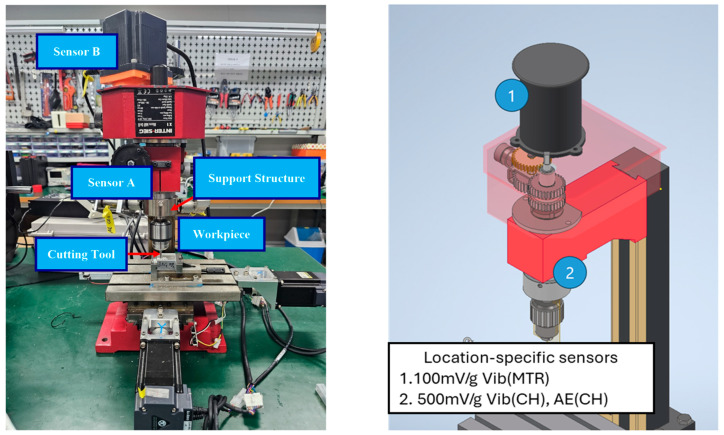
Experimental setup displaying the milling machine equipped with vibration sensors.

**Figure 8:**
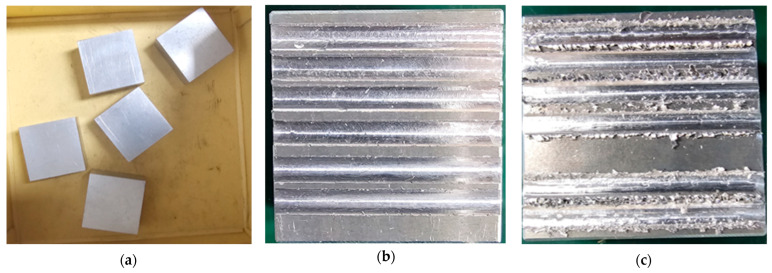
Samples used in the study: (**a**) Workpiece samples, (**b**) unprocessed workpieces, (**c**) workpieces after the milling process.

Vibrational signals were acquired from a real-world milling machine, as shown in Figure 7. The machining center used for this study was an INTER-SIEG X1 Micro Mill Drill, a compact cast iron machine with capabilities like a small pillar drill. The data collection process focused on a straight parallel-milling operation on a steel workpiece, a commonly used techniques for shaping and machining hard materials. Five steel workpieces, each with dimensions of 20 mm × 35 mm × 35 mm, were used for the experiment. Figure 8a depicts the raw workpieces, while Figure 8b,c displays a machined work-piece post-processing. Vibrational sensors (model R15I-AST, MISTRAS Inc., Princeton Junction, NJ, USA) were securely mounted on the milling machine using adhesive to ensure stability during operation. Data acquisition was conducted using the NI-9223 system from National Instruments, with custom software developed in Python 3.10 by the Ulsan Industrial Artificial Intelligence Laboratory. The vibrational signals were sampled at a frequency of 2.56 KHz, providing 256,000 data points per second. Prior to data acquisition, an HSU-Nelson test was performed to verify the proper functioning of the vibrational sensors and to ensure accurate detection of signals during the experiments. To enhance data quality, two vibrational sensors were deployed. The primary sensor was positioned on the spindle to capture critical signals associated with the tool, bearings, and gears, while the secondary sensor acted as a guard transducer. The guard transducer, illustrated in Figure 7, was designed to detect non-target signals, suppress noise, and ensure that the primary sensor focused exclusively on relevant data. This dual-sensor configuration ensured the collection of high-quality vibrational data, which served as the basis for subsequent fault detection and classification tasks. Data acquisition commenced under the normal operating conditions of the milling machine.

**Figure 9:**
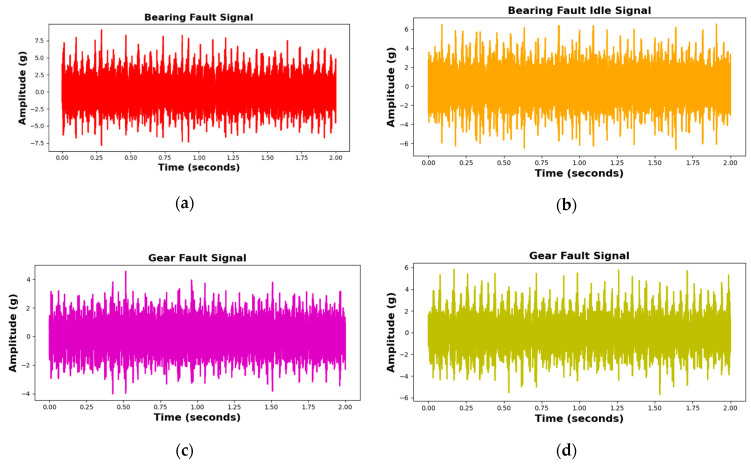

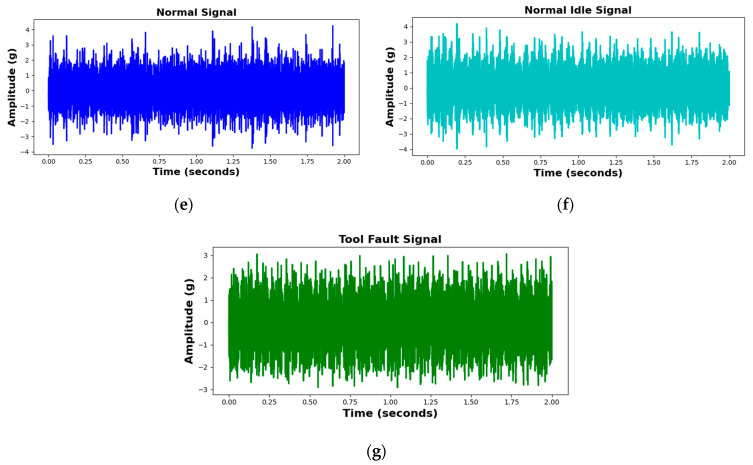
Vibration TD signals of various fault classes: (**a**) BF, (**b**) BFI, (**c**) GF, (**d**) GFI, (**e**) N, (**f**) NI, and (**g**) TF.

In accordance with ISO-8688-2 guidelines [44], the tool’s lifespan was defined by an average flank wear of 0.3 mm. However, tool breakage, which can occur unpredictably during the machining of hard materials, poses a significant risk of catastrophic failure even in the early stages of tool wear. To ensure the collection of fault data for this study, an average flank wear of 0.3 mm was deliberately induced in the carbide tool prior to the milling operation. In addition, to simulate incipient defects, a fault was introduced in the outer race of the bearing supporting the tool, and vibrational signals were recorded during the milling process to capture relevant fault data. A further defect was created in the gear mechanism responsible for torque transmission from the motor to the spindle by removing a small metal fragment from one of the gear teeth. This defect was carefully monitored, and vibrational signals were collected throughout the machining operation to ensure the inclusion of fault-related data. Furthermore, signals overlapping techniques have been used and the samples of each class of the dataset are made 60 as shown in Table 3. These artificially induced defects in the tool, bearing, and gear ensured the dataset comprehensively represented a range of operating conditions, enabling effective fault detection and classification with the proposed method. The details of the dataset are summarized in Table 3 and illustrated in Figure 9.

#### 3.1.1. Milling Cutting Tool Dataset

**Figure 10:**
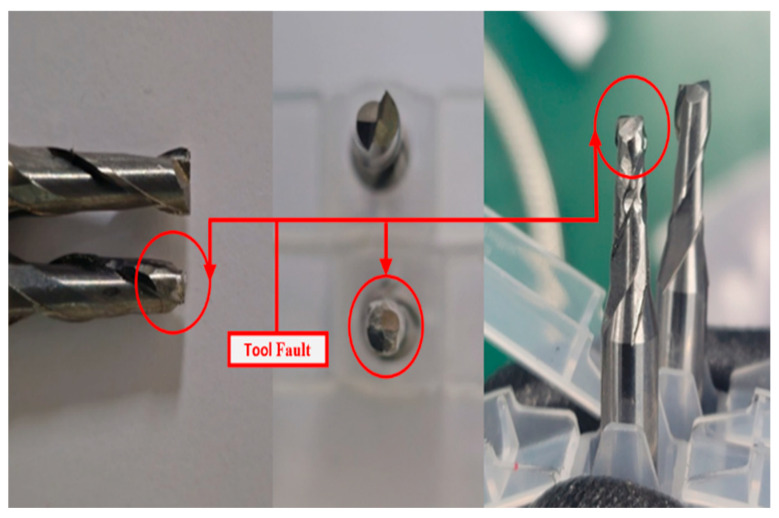
The tool faulted during the experiment.

The MCT Dataset, collected from the MCT testbed, provides comprehensive insights into the health and performance of various machinery components. The experimental setup includes a motor-driven shaft with 16-tooth, 32-tooth, and 30-tooth gears, and a spindle connected to the 30-tooth gear shaft, with a two-flute end mill cutting tool simulating real-world machining. Faults were introduced via laser processing and impact, resulting in three types: 3 mm deep bearing faults, gear faults caused by tooth impact damage, and tool faults from blade breakage as shown in Figure 10. Data was collected under normal and fault conditions during idle and cutting operations. Normal idle conditions involved 15 repetitions of 2-s intervals at 1320 RPM (motor) and 660 RPM (spindle), while cutting conditions used the same speeds and a bed feed rate of 0.4 mm/s. Fault scenarios included 20 idle runs and 15 cutting runs for bearing and gear faults, and 15 cutting runs for tool faults. Vibration signals were sampled at 25,600 Hz using sensors with sensitivities of 100 mV/g for the motor and 500 mV/g for the spindle chuck. Each 2-s segment was recorded via an NI 9234 analog input module with four IEPE channels. This dataset offers valuable information on machinery behavior under diverse conditions, supporting accurate fault detection and classification.

#### 3.1.2. Bearing Dataset

**Figure 11:**
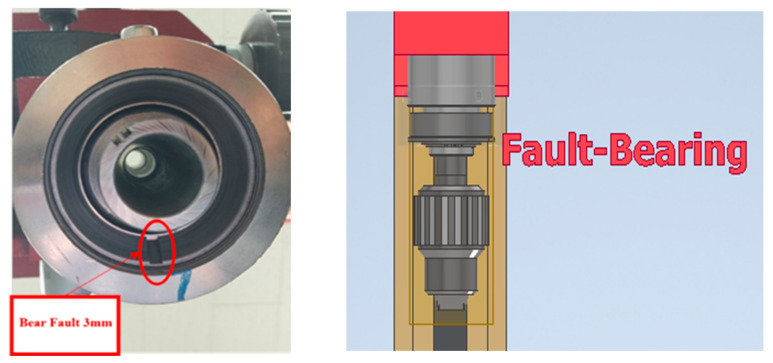
Bearing fault during the experiment.

The Bearing Dataset, sourced from the RK-4 Bearing Testbed, provides extensive information on bearing health conditions. The experimental setup includes a fault simulation testbed with a vibration sensor mounted on the bearing housing for data collection and a tachometer sensor for measuring rotational speed. Data were recorded for both normal and fault conditions, including outer race faults, inner race faults, and roller faults, achieved by replacing the respective bearing components. FAG NJ206TVP rolling element bearings with dimensions of 30 mm inner diameter, 62 mm outer diameter, and 16 mm race width were used in the experiments. Faults were introduced via electrical discharge machining, creating defects with a uniform length of 3 mm and depth of 1 mm as shown in Figure 11.

The bearings operated at 1800 RPM under radial load, and vibration signals were sampled at 25.6 kHz using an IEPE acceleration sensor (model 622B01). Data were collected in 1-second intervals through an NI 9234 four-channel IEPE analog input module. The dataset comprises four bearing health states—normal, inner race fault, outer race fault, and roller fault providing high-resolution data for accurate fault detection and diagnosis.

#### 3.1.3. Gear Dataset

**Figure 12:**
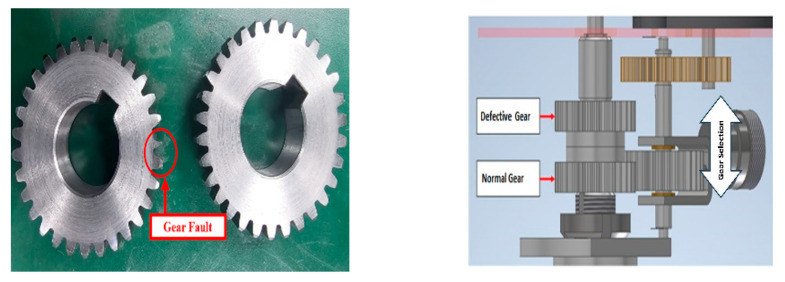
Gear fault during the experiment (milling cutting).

The gear dataset, derived from the Gear Fault Testbed, provides comprehensive data on gear health under varying fault severities and operational conditions. The experimental setup includes a three-phase motor, a gearbox, and a fan connected via a belt to apply directional torque. Gear faults were introduced using laser cutting on the driven shaft, resulting in tooth wear severities of 0.45 mm (5% of tooth length), 0.9 mm (10%), 1.8 mm (20%), 2.7 mm (30%), 3.6 mm (40%), and 4.5 mm (50%). The driven shaft gear features 38 teeth with a diameter of 160 mm, while the drive shaft gear has 25 teeth with a diameter of 108 mm, and each tooth measures 9 mm in length as shown in Figure 12.

Data was captured under rotational speeds of 300 RPM (5 Hz), 600 RPM (10 Hz), 900 RPM (15 Hz), and 1200 RPM (20 Hz), with torque applied through the fan. Vibration signals were recorded at a sampling frequency of 65,536 Hz using an IEPE sensor (model 622B01) for 10-min durations, segmented into 1-s intervals. Sensor placements included Channel 1 on the drive shaft and Channel 2 on the non-drive shaft, providing thorough coverage of gear health states. This dataset offers valuable insights into gear wear progression and fault diagnosis across varying severities and operating speeds.

### 3.2. Performance Metrics for Comparisons

This study presents a fault diagnosis framework integrating ViTs and CNNs to address limited datasets and noisy environments. Using advanced preprocessing, feature fusion, and knowledge distillation, the framework enhances fault detection and classification. Final classifications are carried out using a stacking ensemble with validation-guided weighted voting on the fused features, yielding high accuracy, reduced misclassification, and scalable performance on real-world vibration data. The model’s effectiveness is evaluated using Equations (15)–(18).

(15) $$Accuracy=\frac{\left(TN+TP\right)}{\left(TP+TN+FP+FN\right)}\times 100\%$$

(16) $$Precision=\frac{TP}{TP+FP}\times 100\%$$

(17) $$Recall=\frac{TP}{TP+FN}\times 100\%$$

(18) $$F1-Score=\frac{2TP}{2TP+FP+FN}=\frac{2\times (Precision\times Recall)}{Precision+Recall}$$

Accuracy, precision, recall, and F1-score are key metrics for evaluating classification models. Accuracy measures positive predictions and recalls gauges the model’s ability to identify actual positives. The F1-score balances precision and recall, offering a single performance metric, especially for imbalanced datasets. Together, they provide a comprehensive assessment of model performance.

### 3.3. Ablation Studies

The ViT model, as introduced by Pinghu Xu et al. [45], leverages self-attention mechanisms to effectively capture global dependencies and complex relationships in data, making it well-suited for fault diagnosis tasks. Evaluated on our lab MCT dataset, the ViT model achieved an accuracy of 93.83%, with precision, recall, and F1-scores of 95.00%, 94.00%, and 94.00%, respectively, as shown in Table 4. While these results demonstrate strong overall performance, certain limitations were evident in distinguishing faults with subtle feature differences. For instance, misclassifications between GF and GFI classes were observed in the confusion matrix (Figure 14), and the t-SNE visualization (Figure 15) revealed overlapping clusters for these classes, indicating challenges in learning fine-grained feature separations. Additionally, the ROC curve (Figure 16) showed strong detection performance with an AUC of 1.00 for most classes, but slight reductions were noted for the N and TF classes, with AUC values of 0.97 and 0.98, respectively. These findings highlight the ViT model’s strengths in capturing global relationships and producing well-separated clusters for most classes, but also its limitations in fully addressing subtle fault variations and optimizing performance with limited labeled data.

The ablation study evaluates both 1D-CNN [46] and CWT-CNN [47] for fault classification. The 1D-CNN model, leveraging sequential feature extraction, achieved 93.67% accuracy with precision, recall, and F1 scores of 93.71% each. While effective, it struggles to distinguish faults with subtle variations, particularly in N, NI, and TF classes, as indicated by overlapping clusters in the confusion matrix (Figure 14) and t-SNE visualization (Figure 15). The ROC curve (Figure 16) shows strong detection capability with an AUC of 1.00 for most classes, though Class N has a slight drop to 0.99, reflecting reduced sensitivity in differentiating normal conditions. Its reliance on local feature extraction limits its ability to capture global fault relationships. In the same manner, the CWT-CNN model, using time-frequency scalograms, achieved 91.48% accuracy, with precision, recall, and F1 scores of 91.72% each. It performs slightly worse than the former 1D-CNN, particularly in handling transient fault variations, leading to misclassifications. A key limitation of this model is the use of CWT instead of log-CWT, which results in uneven time-frequency resolution, potentially reducing the model’s ability to capture fine-grained fault patterns. Additionally, the relatively small dataset limits deep learning models from effectively learning complex fault characteristics, impacting classification robustness, especially for closely related fault classes. By integrating ViTs with CNNs in the proposed model, we address these limitations by combining global and local feature extraction, improving class separability, and enhancing robustness in noisy environments. The hybrid approach ensures superior fault classification accuracy and reliability, particularly in challenging scenarios with limited labeled data and overlapping feature distributions.

### 3.4. Comparison of Proposed with Traditional Methods

The proposed model processes input log-CWT scalograms through a ViT architecture for global feature extraction as part of a hybrid fault diagnosis pipeline. The input images, sized (3, 224, 224), are divided into non-overlapping 16 × 16 patches and projected into 384-dimensional embeddings via a patch embedding layer. Positional encodings are added to preserve spatial information across the sequence. These tokenized embeddings, along with a learnable classification token, are fed into 12 sequential transformer blocks comprising multi-head self-attention and MLP layers, enabling the capture of long-range dependencies and global contextual relationships. Instead of direct classification, the output from the classification token is extracted as a compact global feature representation. This is subsequently fused with spatial features extracted from ResNet-18 and forwarded to a stacking ensemble classifier, which generates the final class predictions across seven fault categories using probabilistic outputs aggregated from multiple base learners.

**Figure 13:**
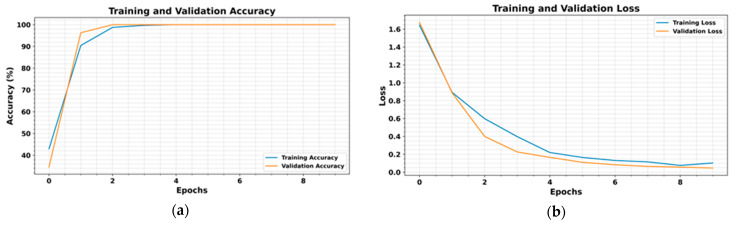
Training and validation (**a**) accuracy and (**b**) losses against the number of epochs.

**Figure 14:**
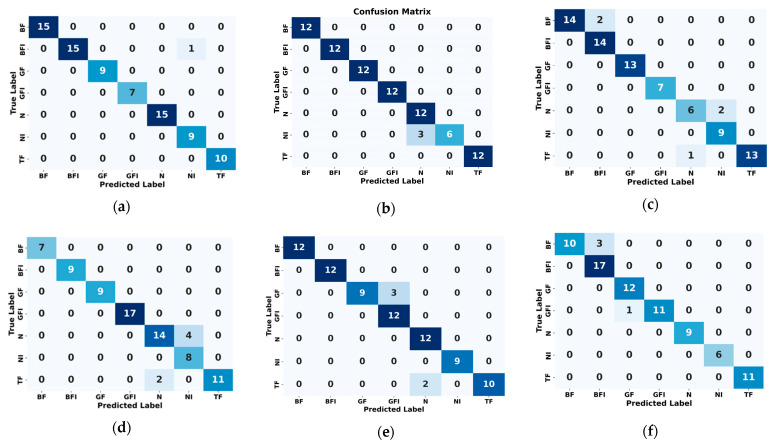
Confusion matrices of the (**a**) Proposed model, with (**b**) CNN-LSTM, (**c**) CNN-CAE, (**d**) ViT(Ablation Study), (**e**) ViT-Attention, and (**f**) 1D–2D CNN fusion.

**Figure 15:**
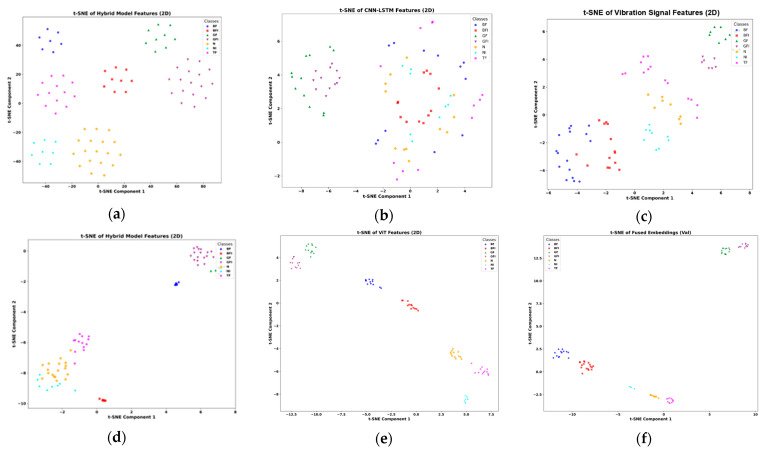
t-SNE comparison of the (**a**) Proposed model, with (**b**) CNN-LSTM, (**c**) CNN-CAE, (**d**) ViT (Ablation Study), (**e**) ViT-Attention, and (**f**) 1D–2D CNN fusion.

**Figure 16:**
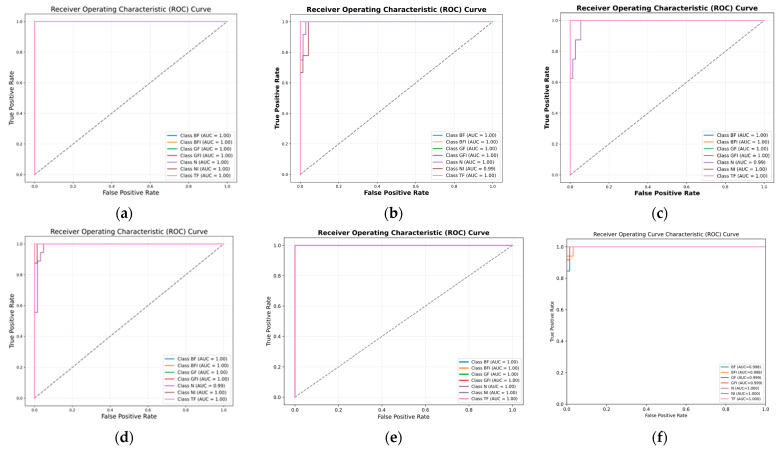
ROC curves comparison of the (**a**) Proposed model, with (**b**) CNN-LSTM, (**c**) CNN-CAE, (**d**) ViT(Ablation Study), (**e**) ViT-Attention, and (**f**) 1D–2D CNN fusion.

The proposed method is validated through comprehensive experiments, demonstrating high classification accuracy and efficiency. As shown in Figure 13, training converges rapidly, with accuracy stabilizing and flattening after just a few epochs. The classification report in Table 5 confirms strong results, with a precision of 98.57%, a recall of 99.14%, and an F1-score of 98.86%, culminating in an overall accuracy of 99.78%. The confusion matrix (Figure 14) indicates minimal misclassifications, with many fault classes correctly predicted. Clear class separability is evident in the t-SNE plot (Figure 15), while ROC curves (Figure 16) confirm excellent detection performance, achieving an AUC of 1.00 across all classes. The integration of ViT and ResNet-18 enables the extraction of both global and local fault features, enhancing the model’s ability to differentiate between subtle fault patterns. Although the results are highly promising, further validation under varied operating conditions is recommended to confirm robustness. Overall, this framework offers a scalable and accurate solution for intelligent fault detection in milling machine applications.

The CNN-LSTM model, proposed by Ting Huang and Dao et al. [48,49] in their studies, achieved 95.29% accuracy, 97.14% precision, 95.29% recall, and 95.57% F1-score on our lab dataset. This hybrid architecture integrates convolutional layers for spatial feature extraction and LSTM layers for capturing temporal dependencies, making it particularly effective for time-series-based fault detection. By analyzing both spatial and sequential patterns, the model aims to enhance fault classification in industrial applications. However, its performance is significantly affected by the limited number of data samples, leading to challenges in distinguishing fault variations. The confusion matrix highlights misclassifications, particularly in the N and NI classes, where three N samples were incorrectly classified as NI, as shown in Figure 13. This suggests that the model struggles with class overlaps and lacks sufficient training data to generalize well across all fault types. The t-SNE visualization, as illustrated in Figure 14, further supports this, showing overlapping clusters for N, NI, and BFI, indicating that the model is unable to create well-separated feature representations due to data scarcity. The ROC curve in Figure 15 shows strong classification performance, with an AUC of 1.00 for most classes, but the N class drops slightly to 0.99, reflecting a minor reduction in sensitivity. While the CNN-LSTM model is designed to capture both spatial and temporal dependencies, its effectiveness is constrained by the limited dataset size, reducing its ability to learn nuanced variations between fault conditions. Enhancing the dataset with more diverse samples and incorporating data augmentation strategies could improve its classification robustness and reliability.

The CNN-CAE model, proposed by Debasish Jana and Zhiyi et al. [50,51] in their respective studies achieved 95.08% accuracy, 95.14% precision, 95.71% recall, and 95.26% F1 score on our lab dataset, as shown in Table 5. Designed for fault classification in industrial systems, it integrates convolutional layers for spatial feature extraction and an autoencoder for dimensionality reduction and noise filtering. This combination allows the model to learn meaningful patterns from complex datasets, making it particularly useful when dealing with inconsistent data quality. However, the model exhibits misclassifications, particularly in the N, NI, and TF classes, as seen in the confusion matrix in Figure 13. These errors suggest difficulties in differentiating closely related fault types, likely due to overlapping feature representations. The t-SNE visualization distinguishes faults with subtle variations, as shown in Figure 14. Additionally, the ROC curve reveals inconsistencies in sensitivity across different classes, with some faults being detected with high confidence while others remain ambiguous, as illustrated in Figure 15. These findings suggest that while CNN-CAE is effective in extracting local spatial features, it struggles to capture global contextual relations. The model’s reliance on localized patterns makes it less capable of handling faults with gradual or subtle changes, which are common in real-world applications. A more advanced approach incorporating global dependencies and context-aware learning mechanisms could improve classification performance across all fault types.

The ViT-Attention model by Lin et al. [52], which integrates convolutional layers with a self-attention mechanism for multi-sensor data fusion, achieved 97.10% accuracy, 97.85% precision, 96.14% recall, and 96.99% F1 score on our dataset, as presented in Table 5. The confusion matrix in Figure 14 confirms that most fault classes were correctly classified, though some misclassifications occurred, particularly between the NI and TF categories. The t-SNE visualization in Figure 15 reveals well-formed but slightly overlapping feature clusters, indicating challenges in distinguishing closely related faults. The ROC curves in Figure 16 show high overall detection confidence, with only minor variations in sensitivity across classes. These outcomes suggest that ViT-Attention is effective in modeling both local and global dependencies but remains sensitive to subtle class overlaps. In contrast, the proposed framework achieves higher robustness and clearer class separation through logarithmic CWT preprocessing combined with a lightweight dual-branch architecture, while avoiding the hardware and computational complexity associated with multi-sensor fusion.

Finally, the 1D-2D CNN Fusion model with hybrid attention proposed by Meng et al. [53] achieved 95.00% accuracy, 96.76% precision, 95.51% recall, and 95.79% F1 score on our dataset (Table 5). As shown in the confusion matrix in Figure 14, most fault types were classified correctly, though some misclassifications occurred between closely related classes such as NI and N. The t-SNE visualization in Figure 15 illustrates reasonable feature clustering, but with minor overlaps that indicate challenges in fully separating subtle fault conditions. The ROC curves in Figure 16 confirm strong sensitivity across all categories, with AUC values approaching 1.00, reflecting reliable detection performance. These findings demonstrate the strength of multi-scale feature fusion combined with hybrid attention in enhancing discriminability and noise resistance. However, architecture remains more complex and computationally demanding than conventional CNNs, which may limit its deployment in real-time or embedded industrial settings. In contrast, the proposed framework achieves comparable or superior performance using a more lightweight dual-branch design, making it more scalable and practical for real-world applications.

### 3.5. Validation of Proposed Model on Public Dataset

**Figure 17:**
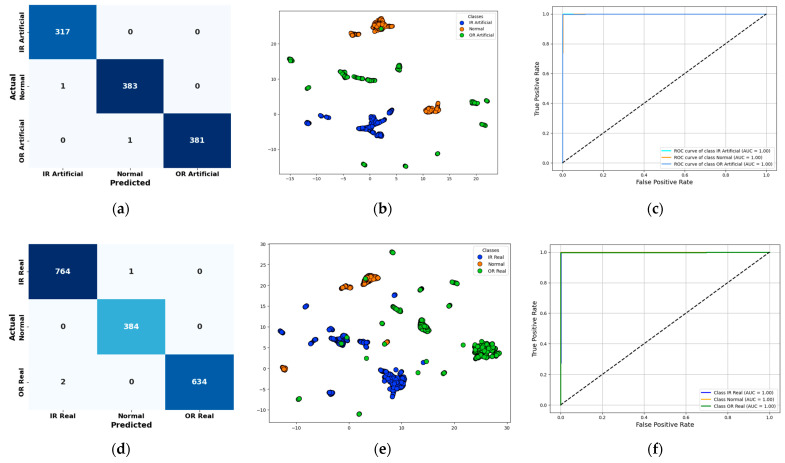
Validation of the proposed model on the Paderborn benchmark dataset: (**a**,**d**) confusion matrices for real and artificial fault data, (**b**,**e**) t-SNE plots showing clear feature separability, and (**c**,**f**) ROC curves demonstrating strong class-wise discriminative performance.

To assess the generalization capability of the proposed model, it was validated using the publicly available Paderborn bearing dataset. The classification report, summarized in Table 5, highlights high accuracy, precision, recall, and F1-scores across all fault categories. The corresponding visual results include confusion matrices Figure 17 for both real and artificial fault data, t-SNE plots Figure 17 illustrating clear feature separability, and ROC curves demonstrating strong class-wise discriminative performance as shown in Figure 17. These results confirm the model’s robustness and reliability on benchmark datasets. To assess external validity, the Paderborn dataset was employed without fine-tuning, using comparable health states (healthy, outer race fault, inner race fault) that conceptually align with the in-house milling dataset. The proposed hybrid model achieved 99.83% accuracy under this cross-dataset evaluation, outperforming CNN-only and ViT-only baselines. This demonstrates the framework’s strong generalization capability across different acquisition setups and operating conditions.

In summary, the proposed ViT–ResNet-18 hybrid model is evaluated through ablation studies and comparative analysis with standalone ViT, 1D-CNN, CWT-CNN, CNN-LSTM, CNN-CAE, ViT-Attention, and 1D–2D CNN fusion architectures. While these individual models demonstrate notable strengths—for instance, CNN-CAE effectively reduces noise via autoencoding, ViT-Attention captures inter-modal dependencies through self-attention, and 1D–2D CNN Fusion enhances discriminability with multi-scale feature integration—they fall short in accurately distinguishing overlapping or subtle fault types, particularly under limited or noisy data conditions. Misclassifications remain evident in closely related categories such as NI, N, and TF, as revealed by their confusion matrices and t-SNE plots. These limitations highlight the need for a unified architecture capable of capturing both detailed local patterns and long-range dependencies, while remaining computationally efficient for deployment. The proposed framework addresses this by combining ResNet-18 for localized spatial features with ViT for global contextual learning, supported by advanced preprocessing techniques such as log-CWT transformation, mean removal filtering, and Canny edge enhancement. Feature fusion produces a rich, discriminative representation, which is classified using a stacking ensemble composed of diverse base learners and a meta-learner to refine predictions. The model’s effectiveness is confirmed not only on real-world MCT datasets but also through validation on the publicly available Paderborn bearing dataset, where it demonstrates consistent fault classification across both real and artificial conditions. In addition to accuracy and robustness, computational efficiency is critical for industrial deployment. The proposed hybrid ResNet-18 + ViT model demonstrates a favorable trade-off between performance and efficiency, with 27.4 M parameters compared to >85 M in pure ViT models, a training convergence time of 28 min (35% faster than ViT-only), and an average inference latency of 12.3 ms per sample. These results confirm the suitability of the framework for near real-time fault diagnosis under practical hardware constraints. Furthermore, the simulation results demonstrate consistent performance across multiple scenarios, including normal and fault conditions of tools, gears, and bearings. At higher spindle speeds, the framework preserved classification accuracy above 99%, showing resilience to dynamic operating conditions. Under artificially introduced noise, accuracy declined slightly (by 1.2%) but remained significantly higher than conventional CNN-based approaches. Ablation studies further confirm that hybrid CNN–ViT fusion outperforms single-model baselines, particularly in separating closely related fault categories such as GF vs. GFI. These findings, together with the comparative evaluation against CNN-CAE, ViT-Attention, and 1D–2D CNN Fusion, validate the scalability, robustness, and practical potential of the proposed framework across a broad spectrum of industrial scenarios. To examine robustness under realistic operating conditions, Gaussian noise was injected into vibration signals at different SNR levels of 30 dB, 20 dB, and 10 dB. The proposed framework maintained excellent performance at 30 dB and 20 dB, with only slight reductions in accuracy and F1-scores relative to clean signals. Even at 10 dB, where noise heavily overlaps with fault signatures, the framework demonstrated stable predictions and significantly outperformed CNN- and ViT-only baselines. These findings highlight that the combination of log-CWT scalograms, Canny edge enhancement, and hybrid dual-branch feature extraction ensures strong resilience to sensor noise and environmental disturbances.

Despite its strong performance, practical deployment of the proposed framework faces several challenges. First, the computational demands of hybrid CNN–ViT models may limit real-time use in resource-constrained environments; lightweight variants, pruning, and knowledge distillation can alleviate this issue. Second, sensor noise and environmental disturbances remain unavoidable in industrial setups; robust preprocessing and adaptive filtering are critical to maintain reliability. Third, hardware limitations such as restricted memory and processing power on embedded systems may affect scalability; solutions include edge computing platforms, hardware accelerators, or FPGA/ASIC-based implementations. Addressing these challenges will be essential for translating the proposed framework from controlled lab settings to real-world industrial environments.

## 4. Conclusions

Milling cutting tools are vital in modern manufacturing, requiring reliable fault diagnosis systems to reduce downtime and ensure product quality. Traditional methods often fall short due to noisy signals, limited labeled data, and the laborious, risky, and costly process of fault data collection. This study proposes a robust fault-diagnosis framework that couples dual-branch deep feature learning with a meta-learning ensemble. Raw vibration signals undergo mean removal, are transformed into logarithmic CWT scalograms, and then are refined with Canny edge filtering to sharpen boundaries and suppress artifacts. A convolutional branch (ResNet-18) and a transformer branch (ViT) extract complementary local and global cues, which are fused into a compact, discriminative embedding. On top of these fused features, three complementary base learners, Linear SVM, Logistic Regression, and a shallow MLP, generate preliminary predictions that are combined by a validation-guided, meta-learned weighted voting module rather than a fixed meta-classifier. The proposed method effectively addresses data scarcity, noise, and computational challenges, achieving 99.78% accuracy on real-world vibration signals. It also adapts well to varying fault conditions and machine components, making it suitable for industrial deployment. An ablation study further validates its performance, showing superior accuracy, precision, recall, and F1-scores compared to 1D-CNN, CWT-CNN, CNN-LSTM, CNN-CAE, ViT-Attention, and 1D–2D CNN fusion models. These findings underscore the framework’s robustness, scalability, and efficiency in complex industrial fault diagnosis tasks.

Despite its performance, the framework has certain limitations that present opportunities for future research. The model validation on diverse datasets and real-time implementation on edge devices for practical use. In future work, this framework can be extended beyond milling machines to other industrial rotating systems, such as pumps, bearings, and gears, to further validate its generalization capability. Additionally, incorporating multimodal sensor fusion (e.g., combining vibration with acoustic emission signals) may improve robustness under complex operating conditions. Lightweight architecture and model compression techniques could also be explored to enable real-time implementation on embedded systems. Investigating self-supervised and semi-supervised learning strategies will help reduce dependence on labeled data, making the approach more practical for large-scale industrial adoption. Finally, resilience against hardware-level disturbances such as sensor degradation and actuator malfunctions will be investigated to further enhance the practical reliability of the framework.

## Figures and Tables

**Table 1:** Features extraction using vision transformer.

Stage	Component	Description	Output Shape
Input	Input Images	(Batch, 3, 224, 224)	(Batch, 3, 224, 224)
Patch Embedding	Patch Embedding	Splits the image into 16 × 16 patches; projects to a 384-dim embedding	(Batch, 196, 384)
Positional Encoding	Positional Embeddings	Adds positional information to patch embeddings	(Batch, 196, 384)
Transformer Blocks	Sequential	Contains 12 transformer encoder blocks with multi-head self-attention and MLP layers	(Batch, 196, 384)
Classification Token	CLS Token	A special token representing the whole image	(Batch, 1, 384)

**Table 2:** Hybrid features fusion and classification.

Stage	Component	Description	Output Shape
Feature Extraction (ResNet-18)	Flatten Layer	ResNet-18 outputs are flattened into a 1D vector.	(Batch, ResNet-18_Feature_Size)
Feature Extraction (ViT)	CLS Token	The ViT CLS token encodes the image as a feature vector.	(Batch, ViT_Feature_Size)
Feature Fusion	Concatenation Layer	Concatenates ResNet-18 and ViT features along the feature dimension.	(Batch, ResNet-18_Feature_Size + ViT_Feature_Size)
Fully Connected Layer 1	Dense Layer	A fully connected layer compresses the combined features to an intermediate size	(Batch, 512)
Activation	ReLU	Applies ReLU non-linearity to the intermediate feature vector.	(Batch, 512)
Fully Connected Layer 2	Dense Layer	Maps the intermediate feature vector to the number of classes.	(Batch, 7)
Activation	Softmax	Converts logits to probabilities for classification.	(Batch, 7)
Output	Class Probabilities	Final output probabilities for 7 classes.	(Batch, 7)

**Table 3:** Data acquisition configuration.

Condition	Number of Samples (VS)	Data Samples (Per Second)	Acquisition Time
BF	60	25,600	2 min
BFI	60	25,600	2 min
GF	60	25,600	2 min
GFI	60	25,600	2 min
N	60	25,600	2 min
NI	50	25,600	2 min
TF	60	25,600	2 min

**Table 4:** Performance metrics of the ablation studies (ViT and 1D CNN) models.

Models	Accuracy (%)	Precision (%)	Recall (%)	F1 Score (%)
**ViT model**	93.83	95.00	94.00	94.00
**1D-CNN**	93.67	93.71	93.71	93.71
**CWT-CNN**	91.48	91.72	91.72	91.72

**Table 5:** Comparison of performance metrics of proposed methods with CNN-LSTM, CNN-CAE, ViT-ttention, and 1D-2D CNN fusion models.

Models	Proposed			CNN-LSTM	CNN-CAE	ViT-Attention	1D-2D CNN Fusion
**Accuracy** (**%**)	99.82	99.83	99.78	95.29	95.08	97.10	95.00
**Precision** (**%**)	99.81	99.83	98.57	97.14	95.14	97.85	96.76
**Recall** (**%**)	99.82	99.85	99.14	95.29	95.71	96.14	95.51
**F1 score** (**%**)	99.82	99.84	98.86	95.57	95.26	96.99	95.79
**Datasets**	**Paderborn Artificial**	**Paderborn Real**	**UIAI MCT**				

## Data Availability

The raw data supporting the conclusions of this article will be made available by the authors on request.

## Nomenclature

MCT	Milling Cutting Tool
ViT	Vision Transformer
ANN	Artificial Neural Network
CNN	Convolutional Neural Network
CAE	Convolutional Autoencoder
ReLU	Rectified Linear Unit
DL	Deep Learning
AE	Acoustic Emission
UIAI	Ulsan Industrial Artificial Lab
